# ED Referral Dramatically Reduces Delays of Initial Evaluation in a French TIA Clinic

**DOI:** 10.3389/fneur.2018.00914

**Published:** 2018-10-26

**Authors:** Nicolas Raposo, Jean François Albucher, Vanessa Rousseau, Blandine Acket, François Chollet, Jean Marc Olivot

**Affiliations:** ^1^Department of Neurology, Toulouse University Medical Center, Toulouse, France; ^2^Toulouse University Neuro Imaging Center, UMR 1214, Toulouse, France; ^3^Toulouse University Medical Center, Clinical Investigation Center, Toulouse, France

**Keywords:** transient ischemic attack, TIA clinic, triage, referral, office-based physician, emergency department, delay of care

## Abstract

**Background:** The risk of recurrent brain infarction (BI) is high within the first hours after a transient ischemic attack (TIA). Emergent, specialized, and tailored patient management in a TIA program reduces the risk of recurrent BI after TIA by 80%. New antithrombotic strategies have been successfully tested within 12 h after TIA onset. We aim to investigate the factors associated with a delay of more than 12 h from TIA onset to evaluation in our TIA clinic.

**Methods:** In consecutive patients evaluated in our TIA clinic from 01/2012 to 11/2013, we prospectively collected delays from onset to arrival, baseline characteristics, discharge diagnosis and recurrent BI at 1 week. Referring pathways were dichotomized between office-based physicians (OBP) and emergency departments (ED). Univariate and multivariate logistic regression were performed.

**Results:** 354 patients were evaluated. Mean (+/– *SD*) age was 61 years (+/−18). Median (IQR) ABCD2 score was 3 (2–4). Median (IQR) delay from onset to evaluation was 8 h (4–48). Overall, 185 (52%) were referred by OBP vs. 169 (48%) by ED. Evaluation was initiated within 12 h among 201 (57%) patients. After logistic regression, OBP referral was by comparison with ED the only independent factor associated with an evaluation delay >12 h (OR 5.7, 95% CI: 3.5–9.3, *p* < 0.0001).

**Conclusion:** Our results suggest that preliminary assessment by OBP may increase the delay to initiate the emergent evaluation of TIA patients. Promoting direct admission to TIA clinics through ED may be an efficient alternative for high risk TIAs.

## Introduction

Up to one third of cases of acute brain infarction (BI) have been preceded by a transient ischemic attack (TIA) (1). The early risk of BI is high after TIA. The risk of invalidating BI after a TIA is major during the first hours following the event (2–4). The emergent, specialized, and tailored management of TIA in the setting of a specific TIA pathway (TIA clinic, clinical decision unit; etc.) reduces the risk of recurrent stroke by 80% (5, 6). Recently, the Platelet-Oriented Inhibition in New TIA (POINT) study demonstrated that the combination of aspirin with clopidogrel therapy initiated within 12 h after TIA onset reduces the risk of recurrent BI by comparison with aspirin alone (7). Therefore, delays in TIA patient evaluation may have a major impact on the risk of recurrent stroke after a TIA.

The emergent management of TIA patients is challenging because the public awareness of the signs of TIA is limited, (8) and the agreement on the clinical diagnosis of the ischemic mechanism of transient neurological symptoms, even by stroke specialist physicians, is low (9).

Two types of medical referral can be distinguished:(10). The first type is Office Based Physician (OBP) referral: The first TIA clinics created in Paris and Oxford were dedicated to accommodating primary care physicians (PCP) to refer TIA patients via a toll free number (5, 6). Hence in Oxfordshire, up to 80% of the patients referred to a TIA clinic did seek medical attention through their PCP first (11). As a consequence, OBP availability (12) as well as their awareness of the risk of stroke after a TIA, (13) could critically influence the delay in patient management. The second type is an Emergency department (ED) referral: in this pathway, patients experiencing TIA symptoms come directly to ED or through a call to the French national medical emergency telephone number (15, equivalent to 911 in the USA). TIA patients are initially evaluated by ER physicians, and then managed by the local stroke team in a clinical decision unit, (14) in a stroke department or referred, after undergoing a predefined work up, to an outpatient TIA program (Two ACES Model) (14–16).

Interestingly, although factors associated with a shorter delay in the management of patients with acute stroke have been identified (17, 18), little is known about the impact of the type of medical referral to a TIA program by OBP vs. ED, or about the delay in initiating emergent comprehensive evaluation of TIA patients.

In 2012, we created a TIA clinic in our stroke unit able to accommodate OBP as well as ED referrals using a toll-free number following the SOS-TIA model (5). Following the same approach we used to monitor IV thrombolysis, during the first 2 years, we collected data on the delays from symptom onset to the initiation of the evaluation by our TIA clinic.

With this as background, we investigated the factors associated with the delay between the symptom onset and the initiation of the evaluation in our TIA clinic using 12 h as a cut off for evaluation.

## Methods

### Patients

Clinical information on consecutive patients referred to our TIA clinic between January, 2012 and November, 2013, were prospectively collected and reviewed. Patients with suspected TIA were referred through a toll free number to our outpatient TIA clinic. The ABCD2 score was dichotomized between < 4 (low risk) and ≥4 (moderate to high risk). Final diagnosis and orientation (hospitalization vs. discharge) and rate of recurrent BI at 1 week were also prospectively recorded. Referral was dichotomized into 2 categories: OBP and ED.

### TIA clinic

Our TIA clinic is composed of 2 monitored beds inside the stroke unit functioning during business hours. At night and during weekends, patients were initially evaluated in the ER then eventually transferred to the unit. When they arrived at the TIA clinic, patients are immediately evaluated by a senior stroke neurologist and underwent, when indicated, emergent MRI, vessel imaging, and expedited complementary work up. Patients were admitted to our stroke unit if they fulfilled admission criteria such as positive DWI lesion on MRI, symptomatic vessel stenosis, recurrent TIA, atrial fibrillation or per the attending stroke neurologist's judgment.

### Statistical analysis

For descriptive analyses, continuous variables are expressed as means and standard deviations (SD) or medians and interquartile ranges (IQR), and categorical variables are expressed as frequencies and percentages. Baseline characteristics were compared according to physician referral type. Factors associated with a delay from symptom onset to admission were compared using multivariate logistic regression model. The delay is categorized into two groups: >12 and ≤ 12 h. Hence, the model estimated the probability of having a delay of medical care of more than 12 h depending on patient characteristics before admission to the TIA clinic. The potential associated factors were: gender (M (ref) vs. F), physician referral (OBP vs. ED), hypertension (yes/no), diabetes (yes/no), dyslipidemia (yes/no), current smoker (yes/no), atrial fibrillation (yes/no), previous stroke or TIA (yes/no), number of TIA episodes (single (ref) vs. recurrent), and ABCD2 score (<4 vs. ≥4 (19)). For the binary factors, the modality “no” was already the reference class. The multivariate model was obtained by including significant associated factors at 30% in univariate models and performing step by step backward elimination. No adjustment was made, i.e., the final model contains only significant factors.

Statistical tests were 2-tailed and were conducted at an alpha level of 5%. Data were analyzed using SAS® software, version 9.4 (SAS Institute).

The study was approved by our hospital research and ethics committee.

## Results

From January 2012 to November 2013, 354 consecutive patients were evaluated by our TIA program. Baseline characteristics are presented on Table 1. Median (IQR) ABCD2 score was 3 (2-4), and 135 (38.1%) had an ABCD2 ≥ 4. Half of the patients (185, 52 %) were referred by an OBP: 129 (70%) by PCP, 2 (1%) by cardiologists, 23 (12%) by ophthalmologists, 31 (17%) others, and 167 (48%) by ED. An MRI was performed on 277 patients (78%) and cervical and intracranial vessel imaging on 306 (86%). An acute DWI lesion was detected in 53 (19%) of these patients, and a symptomatic stenosis in 16 (5%) patients. The final discharge diagnosis from the attending stroke neurologist was TIA or minor stroke in 208 (59%) patients and non-ischemic event in 146 (41%) patients. At the end of evaluation 25% of the patients were admitted to the stroke unit.

**Table 1:** Clinical and Radiological characteristics of the patients.

	**Overall *N* = 354**	**Evaluation initiated within 12 h *N* = 201**	**Evaluation beyond 12 h *N* = 153**	***P*-value**
Age Mean (+/–*SD*)	61.2 ± 17.9	60.1 ± 18.6	62.5 ± 16.9	0.28^x^
Female, *n* (%)	176 (49.7%)	95 (47.3%)	81 (52.9%)	0.29
ABCD2, Median (IQR)	3 (2–4)	3 (2–4)	3 (2–4)	0.06^x^
ABCD2≥4, *n* (%)	135 (38.1%)	87 (43.3%)	48 (31.4%)	0.02
Delay from onset to evaluation, Median (IQR)	8 (4–48)	4 (2–6)	48 (24–96)
Referral type
Office Based Physician *n* (%)	185 (52.3%)	73 (36.3%)	112 (72.2%)	< 0.0001
Emergency Medical Services, *n* (%)	169 (47.7%)	128 (63.7%)	41 (26.8%)
Number of TIA episodes (>1), *n* (%)	60 (17.8)^+^	27 (14.1%)	33 (22.6%)	0.04
Hypertension *n* (%)	144 (40.7%)	79 (39.3%)	65 (42.5%)	0.55
Diabetes, *n* (%)	34 (9.6%)	17 (8.5%)	17 (11.1%)	0.40
Hypercholesterolemia, *n* (%)	109 (31.3%)^*^	57 (28.9%)	52 (34.4%)	0.27
History of stroke or TIA, *n* (%)	55 (15.8%)^*^	33 (16.8%)	22 (14.6%)	0.58
History of MI, *n* (%)	21 (6.0%)^*^	10 (5.1%)	11 (7.3%)	0.39
Atrial fibrillation, *n* (%)	15 (4.2%)	9 (4.5%)	6 (3.9%)	0.80
MRI performed, *n* (%)	277 (78.3%)	159 (79.1%)	118 (77.1%)	0.65
Positive DWI lesion, *n* (%)	53 (19.1%)^°^	33 (20.8%)	20 (16.9%)	0.43
Cervical & intracranial vessel imaging, *n* (%)	306 (86.4%)	176 (87.6%)	130 (85.0%)	0.48
Symptomatic stenosis/occlusion, *n* (%)	16 (5.2%)^$^	13 (7.4%)	3 (2.3%)	0.05
Discharge Diagnosis, TIA or minor stroke, *n* (%)	208 (58.8%)	117 (58.2%)	91 (59.5%)	0.81
Recurrent stroke at 1 week	3 (0.9%)	3 (1.5%)	0 (0.0%)	0.26^#^

* *6 (1.7%) missing data: 4 (2.0%) in 12 h and 2 (1.3%) after 12 h*.

° *Among patients with MRI performed*.

$ *Among patients with Cervical & intracranial vessel imaging*.

X *Wilcoxon Test*.

# *Fisher Test*.

+ *16 (4.5%) missing data: 9 (4.5%) in 12 h and 7 (4.6%) after 12 h*.

By comparison with patients referred by ED, patients referred by OBP tended to have an ABCD2 score <4 (68 vs. 56%, Chi2 Test: *p* = 0.02).

**Figure 1:**
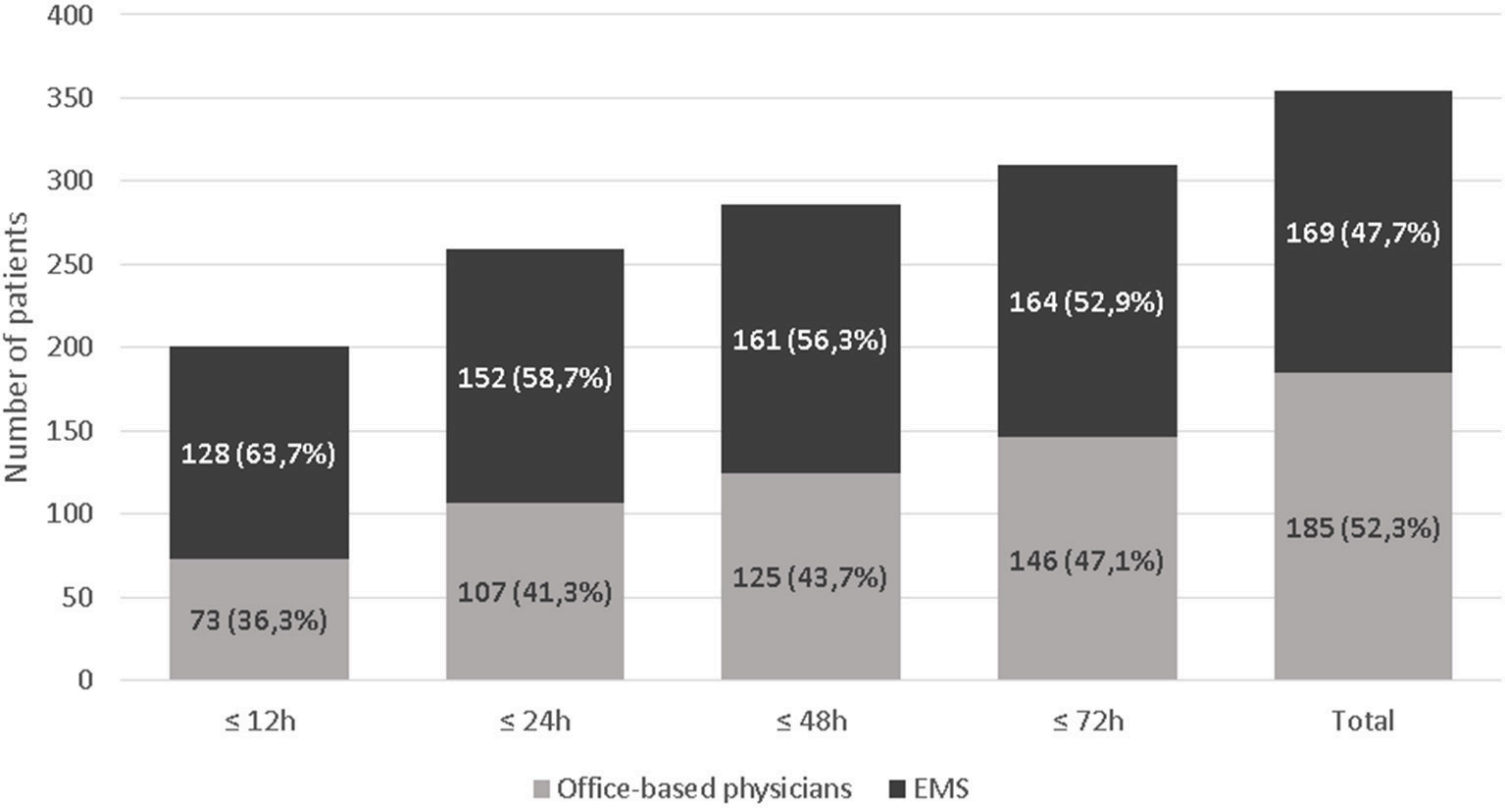
Proportion of patients evaluated within 12, 24, 48, and 72 h according to the type of medical referral.

Median (IQR) delay from onset to arrival in TIA clinic was 8 h (4–48). Initial evaluation was initiated within 12 h after symptom onset in 201 patients (57%). Figure 1 shows the distribution of this delay.

Univariate analysis showed that OBP referral (*p* < 0.0001), number of TIA episodes (*p* = 0.04) and ABCD2 score < 4 (*p* = 0.04) were associated with a delay between onset to evaluation >12 h. After multivariate analysis, referral type (ED vs. OBP) was the only factor associated with the delay from onset to arrival (OR 5.7, 95% CI [3.5–9.3]; *p* = 0.0001).

In the subgroup of 286 (81%) patients evaluated ≤ 48 h after TIA, only OBP referral was associated with a delay >12 h (OR 10.7, 95% CI [5.0–26.5]; *p* < 0.0001).

## Discussion

Our results suggest that in our TIA program, the type of initial medical referral to a TIA clinic critically influences the delay of the initial evaluation.

Baseline characteristics of the patients referred to our TIA program, e.g., median ABCD2 score, DWI positive results, rate of symptomatic vessel stenosis, were in keeping with the results observed in other datasets. By the end of their evaluation, 25% of patients were admitted to the stroke unit. Finally, the rate of recurrent stroke at 1 week was 1.8%. Altogether, those results are in keeping with the findings of the TIA registry published in 2016 (2).

Our results show that in our region, OBP remains the most common type of patient referral. Two thirds of referring OBP were general practitioners. Typically, OBP had limited access to the resources required for the emergent comprehensive evaluation of TIA patients. Therefore, TIA clinic programs were initially designed 15 years ago to expedite the evaluation of these patients (5, 6). Conversely, almost half of the patients were referred by ED. Two thirds of them were referred by the ED of our hospital. These patients were typically directly triaged to our TIA clinic by the triage nurse upon arrival in our ED.

Interestingly, recurrent TIA was more frequent in the subgroup of patients evaluated >12 h after onset. In line with previous studies, these findings suggest that patient awareness of the signs of TIAs does play an important role in delay (11, 20). This relationship did not persist in multivariate analysis. However, this finding suggests that patient awareness of TIA symptoms does play a critical role in delays for initial evaluation. Unfortunately, in the absence of a regional registry, we do not know how many patients did experience a brain infarction after being evaluated in our TIA clinic. In addition, our program, and other similar programs, do evaluate patients who have experienced TIA symptoms within the past few days. We hypothesize that such patients were more prone to visit their PCP rather than contacting ED. In order to address this bias, we restricted our analyses to the subgroup of patients who were initially evaluated by OBP and ED within 48 h after TIA, and again, OBP referral was the only independent factor associated with a delay>12 h for initiating evaluation in the TIA clinic.

Recently, intensive prevention strategies have shown promising results (SOCRATES trial, (21) POINT trial (7)) or are currently (Thales trial; ClinicalTrials.gov: NCT03354429) tested within the first hours after high risk TIA/minor stroke onset defined by simple criteria: such as transient/recurrent/persistent speech disturbance and/or arm weakness in patients with high risk condition (atrial fibrillation, vessel stenosis). Our results suggest that triaging such patients by ED may increase the rate of patients who will receive such emergent treatment within the recommended time window. Interestingly, in our dataset patients referred by ED were more likely to have some of those characteristics such as an ABCD2 score >4 suggesting that some of them may be easily implemented for the triage of such patients. However, several studies have shown that a significant proportion of patients with low ABCD2 score and/or unusual TIA symptoms are still at a high risk of recurrent BI and should therefore be rapidly evaluated in a TIA clinic (22). ABCD2 score accuracy for predicting the risk of stroke after a TIA has been shown to be limited compared to DWI lesion or vascular stenosis or occlusion (4, 23–26).

Our study suffers from several limitations. First the retrospective data collection from a single TIA clinic, and the absence of systematic TIA and stroke registry in our region, create a selection bias and only reflects the characteristics of the patients that were eventually evaluated though our TIA program. Second, our dataset was collected 4 years ago, and since then our program has merged with another similar TIA program, which is currently being, remodeled to incorporate the evolution of TIA management and enrollment into randomized trials. Information campaign and national and European recommendations are currently written to incorporate new aspects of TIA management discussed above.

A new evaluation from larger datasets such as the TIA registry, including other types of TIA programs in other countries, is essential.

The creation of a specific TIA program has been a breakthrough for stroke prevention in the past decade. The initiation of new specific strategies for high risk TIA/minor stroke is now one of the next major challenges for acute phase stroke management. Early identification of high risk TIA/minor stroke patient by ED and transfer to a dedicated structure appears to be an essential step in facing this challenge. Even when they are referred by ED, a number of patients with TIA symptoms experience delays in the management in the TIA clinic, suggesting that information campaigns to the general public on the symptoms of TIA and the early risk of stroke after a TIA are urgently needed.

## Conclusion

In the era of expedited pathways for the evaluation and management of TIA patients, triage by ED results in more expeditious evaluation compatible with emergent therapeutic strategies currently tested to prevent brain infarction after TIA/minor stroke.

## Ethics statement

Our manuscript has been reviewed and approved by our local Clinical Research Comitee.

## Author contributions

NR, JA, FC, BA, and JO acquired data. NR and JO conceived the study. JO drafted the manuscript. VR performed the statistical analysis. NR, JA, FC, and VR reviewed the manuscript.

### Conflict of interest statement

JA received consulting fees from Bayer and speaker honoraria from Boehringer Ingelheim, Bayer and Pfizer. FC served as a consultant for Institut de Recherche Pierre Fabre and has received speaker honoraria from Bristol-Myers Squibb and Boston Scientific. JO received consulting fees from AstraZeneca, Servier, and Medtronic and speaker honoraria from Pfizer and Bristol Myers Squibb. The remaining authors declare that the research was conducted in the absence of any commercial or financial relationships that could be construed as a potential conflict of interest.

## References

[1] Rothwell PM Warlow CP. Timing of TIAs preceding stroke: time window for prevention is very short. Neurology (2005) 64:817–20. 10.1212/01.WNL.0000152985.32732.EE 15753415

[2] Amarenco P Lavallee PC Labreuche J Albers GW Bornstein NM Canhao P. One-year risk of stroke after transient ischemic attack or minor stroke. N Engl J Med. (2016) 374:1533–42. 10.1056/NEJMoa1412981 27096581

[3] Chandratheva A Mehta Z Geraghty OC Marquardt L Rothwell PM. Oxford Vascular S. Population-based study of risk and predictors of stroke in the first few hours after a TIA. Neurology (2009) 72:1941–7. 10.1212/WNL.0b013e3181a826ad 19487652 PMC2690971

[4] Hill MD Yiannakoulias N Jeerakathil T Tu JV Svenson LW Schopflocher DP. The high risk of stroke immediately after transient ischemic attack: a population-based study. Neurology (2004) 62:2015–20. 10.1212/01.WNL.0000129482.70315.2F 15184607

[5] Lavallee PC Meseguer E Abboud H Cabrejo L Olivot JM Simon O. A transient ischaemic attack clinic with round-the-clock access (SOS-TIA): feasibility and effects. Lancet Neurol. (2007) 6:953–60. 10.1016/S1474-4422(07)70248-X 17928270

[6] Rothwell PM Giles MF Chandratheva A Marquardt L Geraghty O Redgrave JN. Effect of urgent treatment of transient ischaemic attack and minor stroke on early recurrent stroke (EXPRESS study): a prospective population-based sequential comparison. Lancet (2007) 370:1432–42. 10.1016/S0140-6736(07)61448-2 17928046

[7] Johnston SC Easton JD Farrant M Barsan W Conwit RA Elm JJ. Clopidogrel and aspirin in acute ischemic stroke and high-risk TIA. N Engl J Med. (2018) 379:215–225. 10.1056/NEJMoa1800410 29766750 PMC6193486

[8] Toole JF. Transient ischemic attack: awareness and prevalence in the community. Health Rep. (1994) 6:121–5. 7919068

[9] Castle J Mlynash M Lee K Caulfield AF Wolford C Kemp S. Agreement regarding diagnosis of transient ischemic attack fairly low among stroke-trained neurologists. Stroke (2010) 41:1367–70. 10.1161/STROKEAHA.109.577650 20508192

[10] Evans BA Ali K Bulger J Ford GA Jones M Moore C. Referral pathways for patients with TIA avoiding hospital admission: a scoping review. BMJ Open (2017) 7:e013443. 10.1136/bmjopen-2016-013443 28196949 PMC5318551

[11] Chandratheva A Lasserson DS Geraghty OC Rothwell PM. Oxford Vascular S. Population-based study of behavior immediately after transient ischemic attack and minor stroke in 1000 consecutive patients: lessons for public education. Stroke (2010) 41:1108–14. 10.1161/STROKEAHA.109.576611 20395614

[12] Lasserson DS Chandratheva A Giles MF Mant D Rothwell PM. Influence of general practice opening hours on delay in seeking medical attention after transient ischaemic attack (TIA) and minor stroke: prospective population based study. BMJ (2008) 337:a1569. 10.1136/bmj.a1569 18801867 PMC2548294

[13] Streit S Baumann P Barth J Mattle HP Arnold M Bassetti CL. Awareness of stroke risk after TIA in Swiss general practitioners and hospital physicians. PLoS ONE (2015) 10:e0135885. 10.1371/journal.pone.0135885 26284533 PMC4540278

[14] Vora N Tung CE Mlynash M Garcia M Kemp S Kleinman J. TIA triage in emergency department using acute MRI (TIA-TEAM): a feasibility and safety study. Int J Stroke (2015) 10:343–7. 10.1111/ijs.12390 25367837

[15] Bravata DM Myers LJ Arling G Miech EJ Damush T Sico JJ. Quality of care for veterans with transient ischemic attack and minor stroke. JAMA Neurol. (2018) 75:419–27. 10.1001/jamaneurol.2017.4648 29404578 PMC5885264

[16] Olivot JM Wolford C Castle J Mlynash M Schwartz NE Lansberg MG. Two aces: transient ischemic attack work-up as outpatient assessment of clinical evaluation and safety. Stroke (2011) 42:1839–43. 10.1161/STROKEAHA.110.608380 21617143

[17] Rossnagel K Jungehulsing GJ Nolte CH Muller-Nordhorn J Roll S Wegscheider K. Out-of-hospital delays in patients with acute stroke. Ann Emerg Med. (2004) 44:476–83. 10.1016/j.annemergmed.2004.06.019 15520707

[18] Bouckaert M Lemmens R Thijs V. Reducing prehospital delay in acute stroke. Nat Rev Neurol. (2009) 5:477–83. 10.1038/nrneurol.2009.116 19668246

[19] Johnston SC Rothwell PM Nguyen-Huynh MN Giles MF Elkins JS Bernstein AL. Validation and refinement of scores to predict very early stroke risk after transient ischaemic attack. Lancet (2007) 369:283–92. 10.1016/S0140-6736(07)60150-0 17258668

[20] Stroebele N Muller-Riemenschneider F Nolte CH Muller-Nordhorn J Bockelbrink A Willich SN. Knowledge of risk factors, and warning signs of stroke: a systematic review from a gender perspective. Int J Stroke (2011) 6:60–6. 10.1111/j.1747-4949.2010.00540.x 21205242

[21] Johnston SC Amarenco P Ticagrelor versus aspirin in acute stroke or transient ischemic attack. N Engl J Med. (2016) 375:1395 10.1056/NEJMoa1603060 27705253

[22] Amarenco P Labreuche J Lavallee PC Meseguer E Cabrejo L Slaoui T. Does ABCD2 score below 4 allow more time to evaluate patients with a transient ischemic attack? Stroke (2009) 40:3091–5. 10.1161/STROKEAHA.109.552042 19520988

[23] Perry JJ Sharma M Sivilotti ML Sutherland J Symington C Worster A. Prospective validation of the ABCD2 score for patients in the emergency department with transient ischemic attack. CMAJ (2011) 183:1137–45. 10.1503/cmaj.101668 21646462 PMC3134721

[24] Kamal N Hill MD Blacquiere DP Boulanger JM Boyle K Buck B. Rapid assessment and treatment of transient ischemic attacks and minor stroke in canadian emergency departments: time for a paradigm shift. Stroke (2015) 46:2987–90. 10.1161/STROKEAHA.115.010454 26316346

[25] Calvet D Touze E Oppenheim C Turc G Meder JF Mas JL. DWI lesions and TIA etiology improve the prediction of stroke after TIA. Stroke (2009) 40:187–92. 10.1161/STROKEAHA.108.515817 18988917

[26] Coutts SB Simon JE Eliasziw M Sohn CH Hill MD Barber PA. Triaging transient ischemic attack and minor stroke patients using acute magnetic resonance imaging. Ann Neurol. (2005) 57:848–54. 10.1002/ana.20497 15929051

